# Study on Viscoelastic Properties of Various Fiber-Reinforced Asphalt Binders

**DOI:** 10.3390/ma17051085

**Published:** 2024-02-27

**Authors:** Yunyu Li, Fan Xu, Yongsheng Wang, Hao Liu, Longfan Peng, Yue Xiao, Qian Liang, Xuquan Li

**Affiliations:** 1 School of Transportation and Logistics Engineering, Wuhan University of Technology, Wuhan 430063, China; 2 School of Naval Architecture, Ocean and Energy Power Engineering, Wuhan University of Technology, Wuhan 430063, China; xufan99@whut.edu.cn (F.X.); lixuquan@whut.edu.cn (X.L.); 3 China Construction Second Engineering Bureau Ltd., Beijing 100176, China; wangyongsheng@cscec.com (Y.W.); liuhao-2@cscec.com (H.L.); penglongfan@cscec.com (L.P.); 4 School of Materials Science and Engineering, Wuhan University of Technology, Wuhan 430070, China; xiaoy@chd.edu.cn; 5 School of Materials Science and Engineering, Chang’an University, Xi’an 710018, China; 6 Department of Power Engineering, Wuhan Electric Power Technical College, Wuhan 430074, China; liangqian1016@163.com

**Keywords:** viscoelastic property, fiber-reinforced asphalt binders, DSR test, discrete time spectrum, master curve

## Abstract

This study analyzed the viscoelastic properties of asphalt binders reinforced with various fibers, such as modified asphalt binder, modified asphalt binder reinforced with lignin fibers (LFs), polyester fibers (PFs), and polypropylene fibers (PPFs), using dynamic shear rheological (DSR) testing. Then, the experiment generated data on the dynamic modulus and phase angle, which described the dynamic rheological characteristics at varying temperatures. The generalized Maxwell model was employed to select the appropriate element, and the test curve was fitted into a discrete time spectrum based on the time–temperature equivalence principle (TTSP). The master curves of the relaxation modulus and creep compliance were established to predict the relaxation and creep properties of various asphalt binders. The analysis indicated that fiber-reinforced binders offer superior resistance to high temperatures and long-term deformation, while being less sensitive to temperature and having a more significant elastic characterization. The binders reinforced with PPFs and LFs exhibited superior performance in high-temperature settings and long-term durability, respectively. On the other hand, the binder reinforced with PFs displayed exceptional high-temperature elastic properties. Additionally, based on the experimental data and corresponding discussion, it appears that the 13-element GM model is more appropriate for fitting the data.

## 1. Introduction

Asphalt mixtures are the most commonly used materials for constructing pavement layers on roads and highways due to their benefits of providing smooth and comfortable driving, low noise, easy maintenance, and recyclability [1,2,3]. With the development of the economy, the high traffic density, heavy loads, and temperature change have led to premature deterioration and inadequate performance of the asphalt pavement structure, resulting in various types of distress such as cracking, raveling, and rutting [4,5,6,7,8,9,10]. 

Many methods have been researched and developed to improve the performance of asphalt pavements, such as modified asphalt technology, Stone Matrix Asphalt (SMA), etc. [11,12,13]. Among the solutions, the most effective and most widely accepted is the addition of fibers to the asphalt as a reinforcement. Crispino et al. [14] found that cellulose-based fibers were beneficial to the compaction properties, the volumetric characteristics, and the failure resistance of bituminous mixtures. Laboratory tests were carried out by Vale et al. [15] to reveal the behavior of coconut fibers and cellulose fibers on SMA mixtures using two different design methods. The results concluded that the addition of fibers in the SMA mixtures can improve tensile strength and fatigue behavior. Direct tension tests and fatigue tests on basalt fiber-reinforced asphalt binder and mastic were conducted by Dong et al. [16] The results showed that by adding appropriate fiber content, an increase in tensile strength, stiffness modules, and fatigue resistance could be achieved. Xiong et al. [17] investigated the reinforcing effects of brucite fibers, lignin fibers, basalt fibers, and polyester fibers on asphalt binding material. Brucite fibers were found to be the most effective in improving the rutting resistance, shear resistance, and dynamic shear modulus of the asphalt matrix when compared to the other fibers. Chen et al. [18] conducted research on the performance of corn straw fiber-modified asphalt binders, and the results showed that their addition can reduce the temperature sensitivity of asphalt binders. The pavement properties of lignin or glass fibers or a composite mixture of both to asphalt mixes were studied by Khater et al. [19]. The optimum bitumen content in asphalt mixes was obtained, while the test results indicated that the addition of fibers to asphalt mixes can improve water damage resistance and low-temperature cracking. The influence of polyester fiber content on the low-temperature crack resistance of permeable asphalt mixture was studied by Wu et al. [20], with conclusions indicating that the addition of a suitable amount of polyester fiber can improve the low-temperature crack resistance due to the reasonable air voids which are critical for flexural tensile strength. Tapkin et al. [21] found that the addition of an appropriate amount of polypropylene fiber had a very positive effect on the properties of asphalt concrete, resulting in good rutting resistance, increased fatigue life, and reduced reflective cracking. Tests on the Marshall stability of polypropylene fiber-reinforced asphalt mixture were conducted by Ramin Bayat et al. [22], which indicated an optimum fiber content for the best reinforcing effect on the performance of the asphalt mixture. Most studies indicate that the addition of fibers to asphalt materials enhances their properties. However, there is rarely research on the effects of different types of fiber on asphalt material properties.

Asphalt binder and the asphalt mixtures made from it are viscoelastic materials whose mechanical properties are significantly affected by temperature [23]. They undergo softening at high temperatures and turn brittle at low temperatures leading to rutting, cracking, and fatigue damage, drastically hindering pavement performance. Therefore, the investigation of viscoelasticity is crucial to understand the constitutive equation, deformation response behaviors, and damage characteristics of asphalt materials, and to help evaluate the field performance of asphalt pavements [24,25]. 

Several researchers have proposed some methods to study the viscoelastic properties of asphalt mixture, mastics, and binder. Based on DSR and MSCR tests, Lagos-Varas et al. [26] proposed a viscoelastic model established by a set of springs and fractional dampers to determine the behavior of asphalt rubber. You et al. [27] tested mineral filler asphalt at high temperatures, using the Brookfield DV-III rotational viscometer and the DSR, which showed that the Maxwell model and the Burgers model can describe the creep characteristics of base asphalt mastic and modified asphalt mastics, respectively. Furthermore, the relaxation function of the Burgers model was feasible to model the relaxation characteristics of base asphalt binder and modified asphalt mastics. Zhang et al. [28] obtained the dynamic modulus and the creep compliance of AC-13. The relaxation modulus was proved to be converted by the generalized Maxwell model using a Prony series with the collocation method and the least squares method. Static creep tests for neat asphalt mixture, compound diatomite and basalt fiber-reinforced asphalt mixture, and styrene–butadiene–styrene-modified asphalt mixture were conducted by Cheng et al. [29]. The generalized Kelvin model and the generalized Maxwell model were found to be superior to the Burgers model in describing the viscoelastic properties of these asphalt mixtures. In order to investigate the rheological behavior of bitumen, Ehsan Behzadfar et al. [30] conducted various tests and theoretically studied continuum constitutive equations for modelling the viscoelastic response. It was found that the relaxation spectrum, which was derived from the generalized Maxwell model, could adequately predict the linear viscoelastic behavior of the bitumen. In order to describe the viscoelastic properties of asphalt mixture more comprehensively, Lyu et al. [31] showed that the relaxation characteristics of asphalt mixes can be fully described over a wider range of times and temperatures by fitting the experimental data using the generalized Maxwell model and the generalized Kelvin model, achieved by using a relaxation master curve [32].

**Figure 1:**
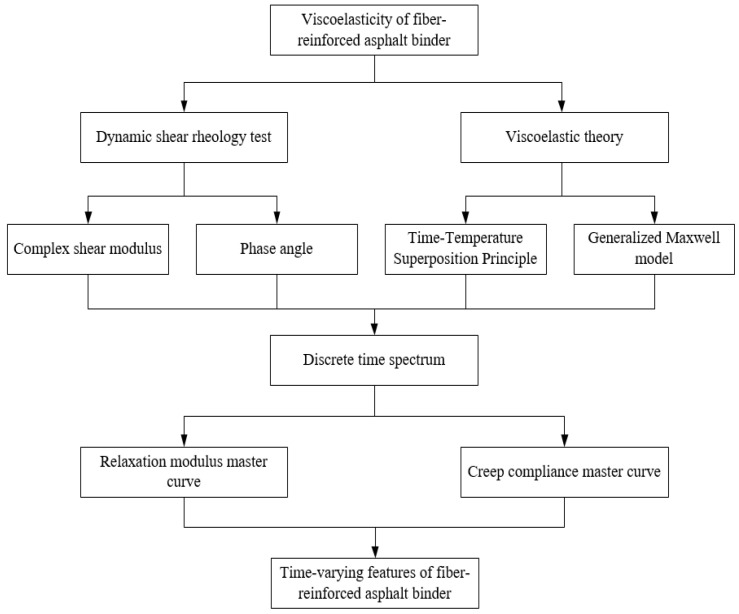
Technical route of this research.

According to the literature mentioned above, research on the viscoelasticity of fiber-reinforced asphalt is limited. The only reported studies have been conducted on asphalt reinforced with lignin fibers. Thus, the aim of this study was to investigate the viscoelastic properties of asphalt binders reinforced with various types of fibers, such as lignin fibers (LFs), polyester fibers (PFs), and polypropylene fibers (PPFs). Furthermore, the study compared the effect of adding these fibers on the viscoelastic characteristics of binders. The study employed Dynamic Shear Rheometer (DSR) tests to examine the dynamic modulus and phase angle at different temperatures. The discrete-time spectra of binders were then determined by analyzing experimental data using the generalized Maxwell model and the time–temperature equivalence principle (TTSP). Finally, the relaxation modulus and creep compliance master curves of binders were depicted by the established optimal constitutive model. In addition, this study examined the long-term deformation resistance of binders through experimental and theoretical methods. The results strongly support the use of fibers in asphalt mixtures and the design of fiber asphalt pavements. The technical roadmap of this study is explained in Figure 1. 

## 2. Materials and Experiment Procedure

### 2.1. Material

#### 2.1.1. Asphalt Binder

In this study, the base asphalt used was the Korean SK Speedway modified asphalt binder, which was tested in the laboratory following the Chinese National Standard (JTG E20-2011) [33]. The specific results of penetration, ductility, softening point, flash point, kinematic viscosity, and solubility are shown in Table 1.

#### 2.1.2. Fibers

Three different types of fiber produced by Hubei Luxiang Chemical Industry (Wuhan, China), namely lignin fibers, polypropylene fibers, and polyester fibers, were used to study the effect of viscoelastic behavior with different fibers at the same dosage on asphalt binders.

**Figure 2:**
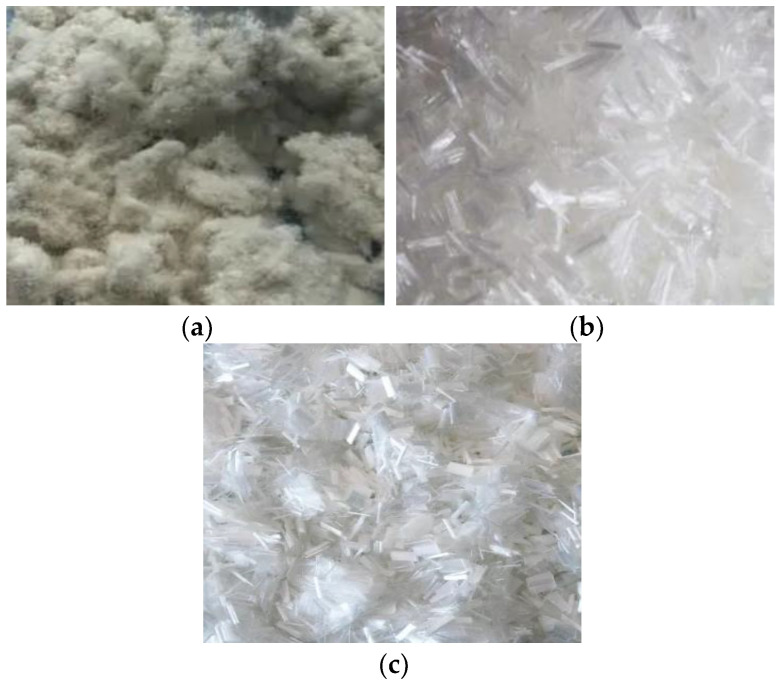
Fibers: (**a**) LFs; (**b**) PPFs; (**c**) PFs.

Lignin fibers (LF) are organic fiber formed within natural wood through complex processing and high-temperature treatment, which appear fluffy and light green. Polypropylene fibers (PPF) are lightweight thermoplastic fibers that appear white in this test. Typically, it is formed through injection molding, extrusion, or blow molding. Polyester fibers (PF) are synthetic, white fibers that are made from the monomers of ethylene glycol and terephthalic acid. The appearance of various fibers is shown in Figure 2.

**Figure 3:**
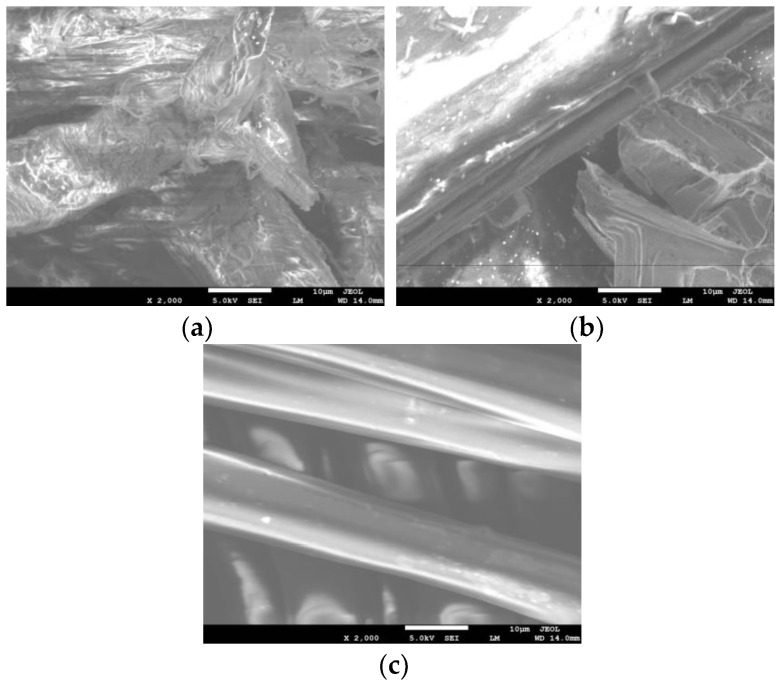
SEM images of fibers: (**a**) LFs; (**b**) PPFs; (**c**) PFs.

To determine the surface structure of the fibers, scanning electron microscopy (SEM) tests were conducted. The results of the test are displayed in Figure 3. The properties of various fibers are presented in Table 2.

The physical properties of fibers, such as tensile strength and density, determine their effectiveness in transmitting stress. To test the tensile strength of the fibers, a constant rate of extension testing machine (CRE) was used. Moisture content was also tested by placing the fibers in a humidor for 120 h, which may affect the water stability of asphalt. In the case of LFs, oil absorption needed to be considered, while for PFs and PPFs, the melting point needed to be taken into account. The test results fulfilled all the requirements of the Chinese asphalt pavement fiber standard (JTT 533-2020) [34], as shown in Table 2.

#### 2.1.3. Mineral Powder

Mineral powder from Wuhan Li Hong Chemical Industry was used in the study and tested according to the Test Methods of Aggregate for Highway Engineering (JTG E42-2005) [35], The apparent density, particle size range, and hydrophilic coefficient were tested, and the test results are shown in Table 3.

### 2.2. Methodology

#### 2.2.1. Preparation of Specimens

**Figure 4:**
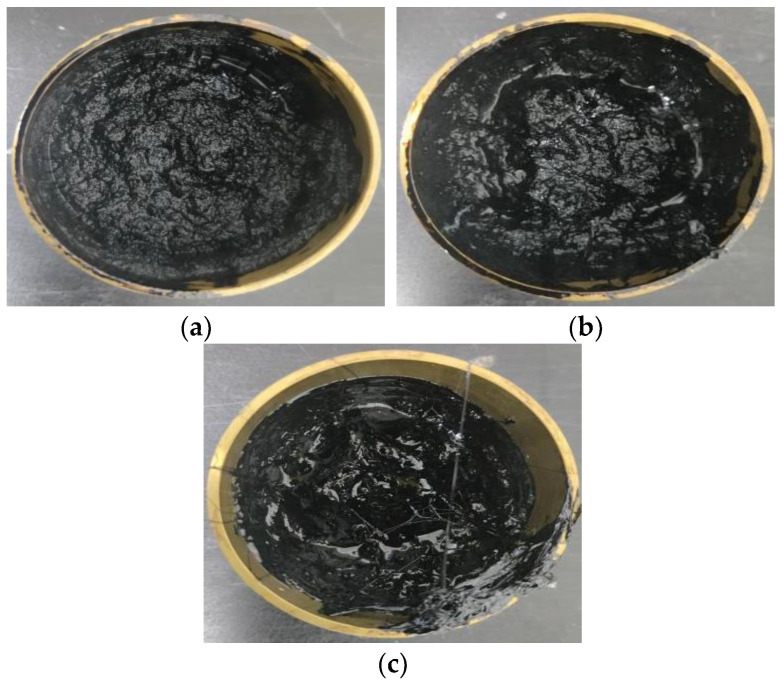
Fiber-reinforced asphalt binders: (**a**) LFs; (**b**) PPFs; (**c**) PFs.

To ensure optimal performance of the modified asphalt binder and prevent separation and precipitation of fibers and mineral powder, preparation in a timely manner was necessary. Firstly, the fibers and mineral powder were dried in an oven set at 120 °C to eliminate moisture. Subsequently, they were slowly added to raw asphalt that had been heated to 155 °C and stirred using a glass rod. An asphalt mixer was then used to mix the above materials at high speed for approximately 1 h to eliminate air bubbles in the mix and ensure that the fiber-reinforced asphalt binders were essentially homogeneous. Finally, the prepared fiber-reinforced asphalt binder specimens were as shown in Figure 4. Moreover, the same method was used to prepare a control group of modified asphalt binder specimens without fiber (the control group was the general modified asphalt binder).

#### 2.2.2. Dynamic Shear Rheology Test

**Figure 5:**
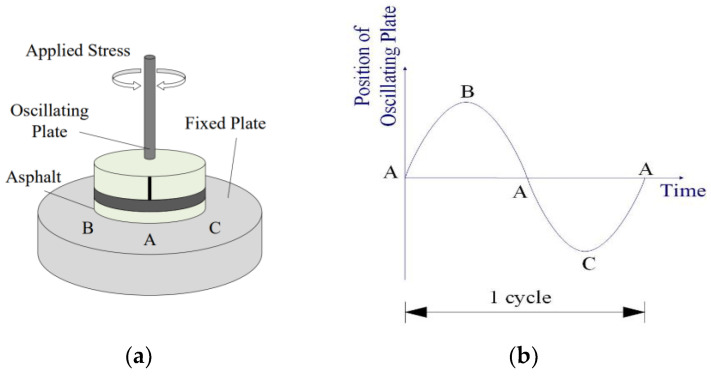
Operational mechanism of the DSR test: (**a**) schematic of DSR testing configuration equipment; (**b**) schematic of the change rule of the position of the oscillating plate with time.

The study used the Dynamic Shear Rheometer (DSR) to measure the complex shear modulus and phase angle of the fiber-reinforced asphalt binder. To do this, the Anton Par Physical MCR-302 was used, which consists of two parallel plates with a diameter of 25 mm—one oscillating plate and one fixed plate—with an asphalt sample placed in between them as shown in Figure 5. To investigate the rheological characteristics of fiber-reinforced asphalt binders, temperature scanning tests with a sinusoidal oscillation load of 10 ± 0.1 rad/s and strain control (5%) were conducted on two groups—one exposed to lower temperatures ranging from −10 °C to 35 °C and the other to higher temperatures ranging from 20 °C to 65 °C. The complex shear modulus G* and phase angle *δ* obtained from the test were used to evaluate the deformation resistance and establish the discrete relaxation spectrum and retardation spectrum.

## 3. Theoretical Background

### 3.1. The Time–Temperature Superposition Principle

Asphalt is a typical rheology material. The Time–Temperature Superposition Principle (TTSP) [36,37] can be employed to determine the viscoelastic master curve for thermal rheological materials in a linear viscoelastic (LVE) manner when subjected to small stress or strain levels. By applying the TTSP, the results obtained from experiments at different temperatures could be horizontally shifted to a certain reference temperature. This process aligns the various curves, ultimately resulting in a single, smooth master curve that predicts material properties on a time and temperature scale larger than that used in laboratory testing. The frequency at the reference temperature in the master curve is defined as the reduced angular frequency *ω*_r_, and can be acquired through Equation (1):

(1) $$\omega_{r}=\omega \alpha_{T}$$

where *ω* = 2π*f* and *f* is the frequency in experiment temperature; *α*_*T*_ is the time–temperature shift factor, which can be calculated based on the Williams–Landel–Ferry (WLF) [38] equation expressed in Equation (2).

(2) $$\mathrm{lg}\alpha_{T}=\frac{-C_{1}\;\pm \;\left(T-T_{r}\right)}{C_{2}+\left(T-T_{r}\right)}$$

where *T* and *T*_r_ are the experimental temperature and the reference temperature, respectively; *C*_1_ and *C*_2_ are the constants for the thermodynamic properties of the material, whose values are dependent on *T*_r_.

### 3.2. Viscoelastic Constitutive Model

The complex modulus obtained from the DSR test consists of two components: the real part and the imaginary part. The real part exhibits elastic properties known as the storage modulus, while the imaginary part represents viscous properties known as the loss modulus. Equation (3) demonstrates the relationships among the parameters:

(3) $${G(i\omega )}^{*}{=G(\omega )}^{\prime }{+\mathrm{iG}(\omega )}^{\prime \prime }$$

where *G*(*iω*)* is the complex modulus; *G*(*ω*)′ is the storage modulus; and *G*(*ω*)″ is the loss modulus. Furthermore, the complex modulus and complex compliance have a reciprocal relationship with each other, as shown in Equation (4):

(4) $$G{\left(i\omega \right)}^{*}=\frac{1}{J{\left(i\omega \right)}^{*}}$$

where *J*(*iω*)* is the complex compliance.

The phase angle indicates the time lag between stress and strain, and can be computed by examining the previous loading cycles. Generally, elastic materials exhibit a phase angle of 0°, while viscous materials show a phase angle of 90°, and for viscoelastic materials the phase angle ranges from 0° to 90° [39]. Moreover, *G*′, *G*″ can be expressed as Equation (5):

(5) $$G^{\prime }=\left|E^{*}\right|\cos\delta ,G^{\prime \prime }=\left|G^{*}\right|\sin\delta ,\tan\delta =\frac{G^{\prime \prime }}{G^{\prime }}$$

where |G*| is the absolute value of the complex modulus, and *δ* is the phase angle.

**Figure 6:**
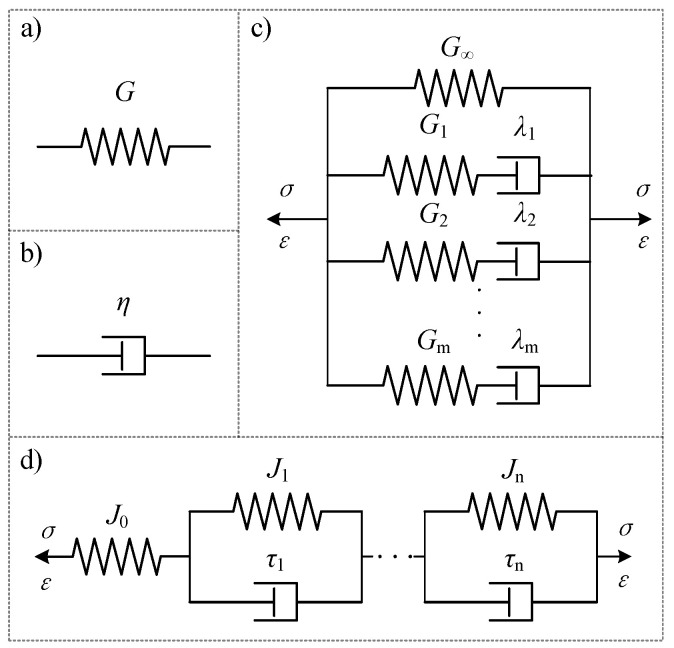
Linear viscoelastic (LVE) models: (**a**) spring element; (**b**) dashpot element; (**c**) generalized Maxwell (GM) model; (**d**) generalized Kelvin–Voigt (GKV) model.

Under the small strain hypothesis, asphalt binders can be considered as a linear viscoelastic material without considering their nonlinear effects. Thus, the viscoelastic response of fiber-reinforced asphalt binders can be described with linear viscoelastic (LVE) models. A number of rheological models with the basic elements of a spring and dashpot have been proposed, such as Maxwell, Kelvin–Voigt, standard linear solids, Burgers, and generalized Maxwell (GM) or Kelvin–Voigt (GKV), among others. Moreover, the GM and GKV models are commonly used to describe asphalt binder’s relaxation and creep properties. The GM model utilizes multiple Maxwell units and a spring element arranged in parallel, whereas the GKV model includes multiple Kelvin units and a spring element arranged in a series. These models are illustrated in Figure 6 and expressed in Equations (6) and (7).

The relaxation modulus of the GM model expressed by the Prony series is shown in Equation (6):

(6) $$G(t)=G_{\infty }+\overset{m}{\underset{i=1}{\sum }}G_{i}e^{-{t/\lambda }_{i}}$$

where *G*(*t*) is the relaxation modulus; *G*_∞_ is the equilibrium modulus over a long period of time, which equals the dynamic modulus with a near-zero frequency reduction; *G*_*i*_ represents the relaxation modulus of the *i*-th Maxwell element in the GM model, while *λ*_i_ is the corresponding *i*-th relaxation time, forming a discrete sequence {*G*_*i*_, *λ*_*i*_} known as the discrete relaxation time spectrum.

The creep compliance of the GKV model expressed by the Prony series is shown in Equation (7):

(7) $$J(t)=J_{0}+\overset{j=1}{\underset{n}{\sum }}J_{j}{(1\;-\;e}^{{(-t/\tau }_{j})})$$

where *J*(*t*) is the creep compliance; *J*_0_ is the instantaneous compliance; *J*_*j*_ represents the creep compliance of the *i*-th Kelvin element in the GKV model; *τ*_*j*_ is the *i*-th’s retardation time, and forming {*J*_*j*_, *τ*_*j*_} as a discrete retardation time spectrum.

Equations (8) and (9) demonstrate the relationship between the parameters of the complex material in both the GM and GKV models. Furthermore, Equation (8) can be used to derive the Prony series for the storage modulus and loss modulus, while storage compliance and loss compliance can be calculated by Equation (9):

(8) $$G^{\prime }(\omega )=G_{\infty }+\overset{m}{\underset{i=1}{\sum }}G_{i}\cdot (\frac{\omega^{2}{\lambda_{i}}^{2}}{{1+\omega }^{2}{\lambda_{i}}^{2}}),\;G^{\prime \prime }(\omega )=\overset{m}{\underset{i=1}{\sum }}G_{i}\cdot \left(\frac{{\omega \lambda }_{i}}{{1+\omega }^{2}{\lambda_{i}}^{2}}\right)$$

(9) $$J^{\prime }{(\omega )=J}_{0}+\overset{n}{\underset{j=1}{\sum }}J_{j}\cdot \left(\frac{1}{\omega^{2}{\tau_{j}}^{2}+1}\right)\;{,\;J}^{\prime \prime }(\omega )=\overset{n}{\underset{j=1}{\sum }}J_{j}\left(\frac{{\omega \tau }_{j}}{\omega^{2}{\tau_{j}}^{2}+1}\right)$$

Substituting Equations (1) and (2) from Section 3.1 into Equation (8) results in Equation (10):

(10) $$G^{\prime }{(\omega )=G}_{\infty }+\overset{m}{\underset{i=1}{\sum }}G_{i}\cdot \left[\frac{{\lambda_{i}}^{2}\omega^{2}\cdot 10^{-\;\frac{{2C}_{1}\left({T-T}_{F}\right)}{C_{2}+\left({T-T}_{T}\right)}}}{1+{\lambda_{i}}^{2}\omega^{2}\cdot 10^{-\;\frac{{2C}_{1}\left({T-T}_{T}\right)}{C_{2}+\left({T-T}_{T}\right)}}}\right]$$

In addition, the storage compliance *J*′ can be calculated by using Equation (11), which utilizes the storage modulus and loss modulus:

(11) $$J^{\prime }(\omega )=\frac{G^{\prime }\left(\omega \right)}{{\left[G^{\prime }\left(\omega \right)\right]}^{2}+{\left[G^{\prime \prime }\left(\omega \right)\right]}^{2}}$$

According to Equation (11), it is feasible to graph the storage compliance master curve, while the discrete retardation time spectrum should fit the storage compliance data according to Equation (9). 

Secondly, the relaxation modulus and creep compliance in the Laplace transform domain satisfy Equation (12):

(12) $$\tilde{G}\left(s\right)\tilde{J}\left(s\right)\;=\;1,$$

where *s* is the Laplace variable.

Based on the identity relation between the relaxation modulus and creep compliance in the Laplace transform domain, *J*_0_ can be derived from Equations (7) and (9), expressing as Equation (13):

(13) $$J_{0}=\lim_{t\to 0}J\left(t\right)=\lim_{s\to \infty }\tilde{J}\left(s\right)=\frac{1}{\lim_{s\to \infty }\tilde{G}\left(s\right)}=\frac{1}{\lim_{t\to 0}G\left(t\right)}=\frac{1}{G_{\infty }+\sum_{i=1}^{m}G_{i}}$$

## 4. Results and Discussion

Temperature influences the viscosity of the asphalt binder. Therefore, the samples of the binders were subjected to DSR testing at different temperatures, with a frequency of 10 ± 0.1 rad/s and a strain control (5%). The resulting complex shear modulus (G*) and phase angle (*δ*) were measured and analyzed to determine the temperature sensitivity of the binders. 

### 4.1. Complex Shear Modulus

**Figure 7:**
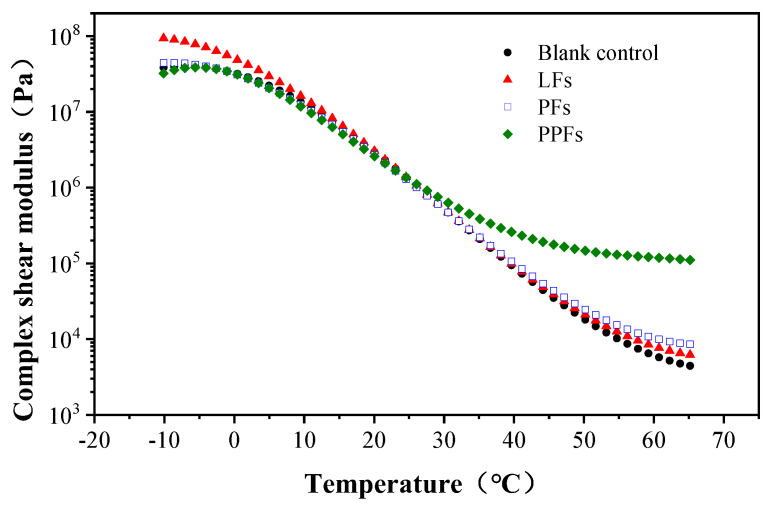
Changing trend of complex shear modulus with temperature.

Figure 7 depicts the pattern of complex modulus G* variation within a temperature range of −10 °C to 65 °C for both general asphalt and fiber-reinforced asphalt binders. The curves indicate that the dynamic modulus decreased with increasing temperature. Moreover, these results demonstrated a temperature sensitivity, as the complex modulus consistently decreased as the temperature increased. In the temperature range of −10 °C to 20 °C, the LF-reinforced asphalt binder exhibited the highest magnitude of complex modulus compared to the others. Furthermore, the G* values for the binders reinforced with fibers were higher compared to that of the general asphalt binder within the temperature range of 20 °C to 65 °C. Notably, among the fiber-reinforced binders, the PPF-reinforced binder exhibited the most significant advantage. Therefore, it can be inferred that the addition of fibers enhances the durability of asphalt binders against deformation during dynamic loading at high and low temperatures. 

### 4.2. Phase Angle

**Figure 8:**
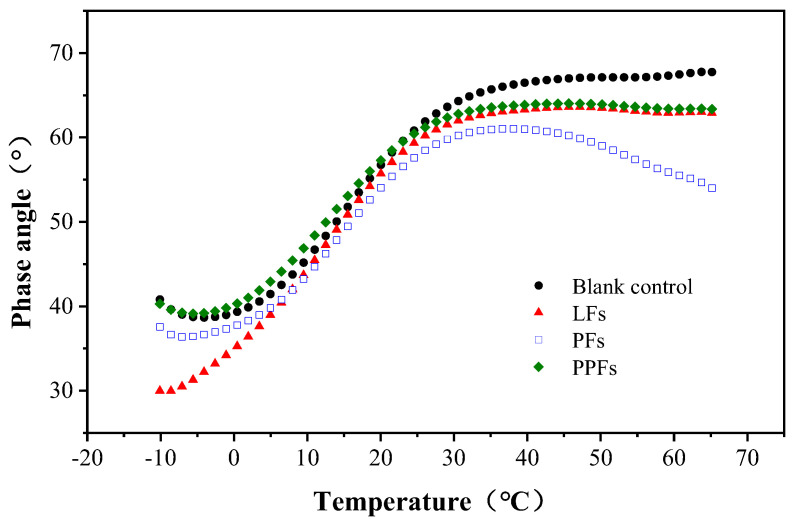
Changing trend of phase angle with temperature.

According to Figure 8, the phase angle *δ* of asphalt binders increased with the temperature, indicating more apparent viscous properties as the temperature rose from −10 °C to 40 °C. At 40 °C, binders reinforced with LFs and PPFs reached their maximum phase angle value and remained stable with increasing temperature. However, the phase angle of the PF-reinforced binder and the general asphalt binder displayed contrasting behavior. In particular, as the temperature increased, the phase angle of the PF-reinforced binder decreased while that of the general asphalt binder steadily increased. In addition, the binder reinforced with PFs demonstrated the lowest phase angle, followed by those reinforced with LFs and PPFs, while the general asphalt binder exhibited the highest phase angle within the temperature range of 20 °C to 65 °C. This is due to the effective adsorption of the asphalt binder by the PFs, which is crucial for stabilizing and enhancing the crack resistance of asphalt. As previously discussed, the presented curves illustrate that fiber-reinforced asphalt binders exhibit lower temperature susceptibility and a more significant elastic characterization when compared to the general asphalt binder.

## 5. Creep Master Curve Development

The master curves of the relaxation modulus and creep compliance can illustrate the time-dependent characteristics of fiber-reinforced asphalt binders. After completing complex modulus tests, the relaxation modulus and creep compliance master curves are typically created using the discrete relaxation spectrum approach. The relaxation spectrum illustrates the distribution of relaxation strength over relaxation time and is essential to the relaxation modulus.

### 5.1. The Discrete Relaxation Time Spectrum and GM Model Analysis

The relaxation (or retardation) time spectrum shows a functional relationship between viscoelastic parameters and time or frequency [40]. This spectrum can establish an exact and dependable viscoelastic model that precisely characterizes the viscoelastic property of asphalt material. In this study, the GM model was used to evaluate the viscoelastic properties of binders using data from testing conducted at a reference temperature of *T*_r_ = 21 °C. Based on the collocation method [41], several relaxation time points were selected as the configuration points. These points were set as *λ*_1_ = 10^5^ and *λ*_*i*_ = *λ*_1_·10^1−*ki*^ (*i* = 2, 3, …, *n*), where *k* denotes the interval of the configuration points on logarithmic coordinates, and was assigned values of *k* = 2, *k* = 1.5 and *k* = 1 corresponding to the unit numbers of the GM model as *m* = 7, *m* = 9 and *m* = 13 respectively. Finally, the discrete values of *E*_*i*_ for the relaxation time spectrum of the general and fiber-reinforced asphalt binders can be determined through the calculation using Equation (10) by employing the setting *λ*_*i*_ and experimental data. 

**Figure 9:**
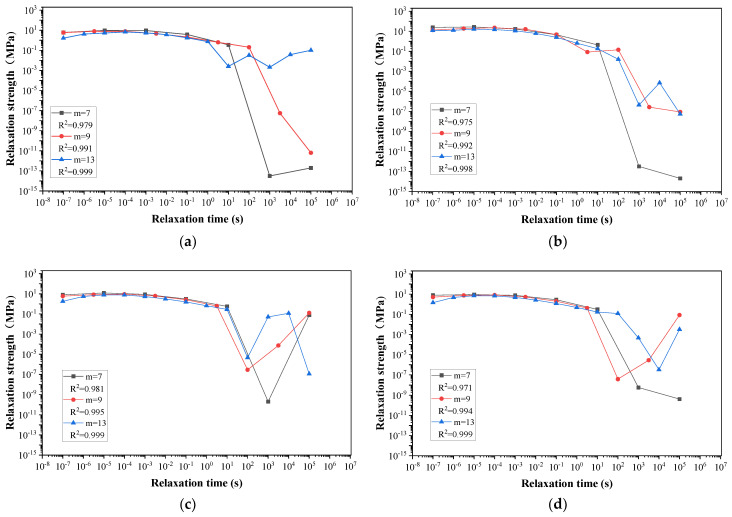
Changing trend of the discrete relaxation time spectrum: (**a**) general asphalt binder; (**b**) LF-reinforced asphalt binder; (**c**) PF-reinforced asphalt binder; (**d**) PPF-reinforced asphalt binder.

Figure 9 demonstrates the discrete relaxation time spectra composed of discrete se-quence {*G*_i_, *λ*_i_} of fiber-reinforced asphalt binders and the general asphalt binder, while the *R*^2^ score acts as an indicator of the goodness of fit for the calculation model. Significant fluctuations were observed in the discrete relaxation time spectrum of all binders when *m* = 7, occurring within the time range of 1 to 10^5^ s. Additionally, the coefficient *R*^2^ exhibited a value below 0.99, demonstrating that the seven-element units GM model inadequately reflected the viscoelastic behaviors of the four asphalt binders. As depicted in Figure 9, the curve with *m* = 13 displays less fluctuation than that with *m* = 9, and the *R*^2^ value for each curve is higher than 0.99. Therefore, it is suggested that utilizing the generalized Maxwell model with 13 elements could lead to enhanced accuracy in calculation and precise depiction of viscoelastic characteristics for each of the four types of asphalt binder. It could be concluded that as the value of *m* increases, greater precision will be achieved. However, this increase also results in a rise in the number of unknown parameters, thus necessitating additional calculations. Therefore, to achieve optimal balance between accuracy and efficiency in calculations, the value of *m* = 13 was selected for this study. 

### 5.2. Establishment of the Master Curves Relaxation Modulus and Creep Compliance

Relaxation and creep are basic characterizations of viscoelasticity, which can be quantified through the relaxation modulus and creep compliance [42]. Creep refers to the reduction in strain over time under constant stress, while relaxation describes the situation where stress decreases over time under steady strain. 

**Figure 10:**
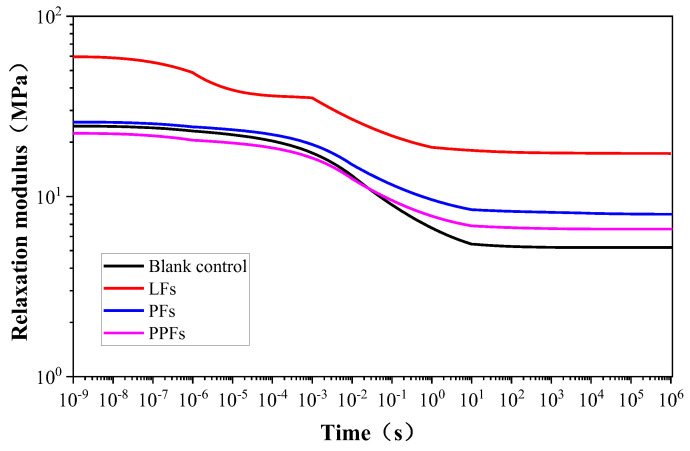
Changing trend of master curves with relaxation modulus.

The master curves at a reference temperature of *T*_r_ = 21 °C for the relaxation modulus of general and fiber-reinforced asphalt binders can be acquired with the employment of Equation (6) and by referring to the relaxation time spectrum featured in Section 4.2. The curves depicted in Figure 10 indicate that all relaxation moduli display a pattern of initial decrease followed by stabilization over time. Moreover, it is noteworthy that the relaxation modulus of the lignin fiber binder exceeds that of the other binders, as indicated by the significant upward deviation solely observed only in the LF curve. In addition, the general asphalt binder, PF-reinforced binder, and PPF-reinforced binder curves demonstrate nearly identical overlap. Upon stabilization, the relaxation modulus for the PF-reinforced binder is ranked first, followed by the PPF-reinforced binder, and finally the general asphalt binder. The data clearly demonstrate that the incorporation of fiber enhances the relaxation modulus of the asphalt binder after stabilization, with lignin fiber producing the most significant increase among the three fiber types. 

**Figure 11:**
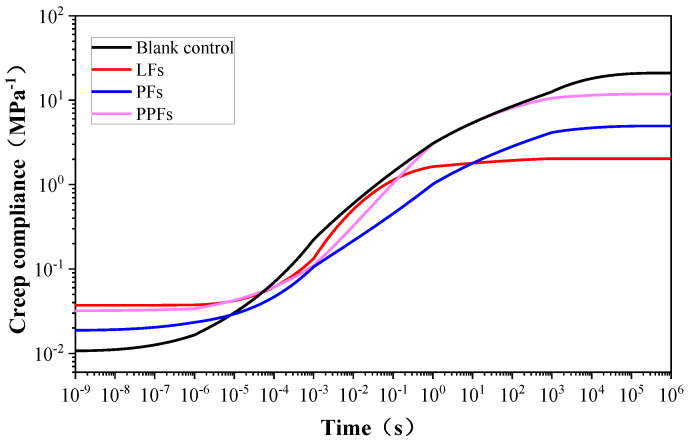
Changing trend of master curves with creep compliance.

Based on the relationship between creep compliance and the relaxation modulus according to Equation (11), the creep compliance master curve can be established, as illustrated in Figure 11. The curves indicate that the creep compliance of all binders gradually increases and eventually stabilizes over time. The stabilization time for creep compliance differs among various binder types. The general asphalt binder exhibited the longest stabilization time, while the other binders reinforced with fibers experienced a shorter stabilization time. Moreover, after stabilization, the general asphalt binder showed the greatest creep compliance, followed by PPF, then PF, and finally LF. The aforementioned statement suggested that the addition of fibers can reduce stabilization time and decrease creep compliance, particularly when incorporating lignin fibers.

A high relaxation modulus and low creep compliance indicate excellent performance in resisting deformation. Hence, the aforementioned discussion of time domain master curves indicated that incorporating fibers is a feasible approach to improving asphalt binders’ capability to withstand deformation in the long term. Of all the types evaluated, lignin fibers exhibited the highest efficacy in this regard.

## 6. Conclusions

The aim of this study was to examine the effect of different fibers, such as LFs, PFs, and PPFs, on their viscoelastic properties. To achieve this, DSR testing was conducted at various temperatures between −10 °C and 65 °C, and the dynamic modulus and phase angle were analyzed. Furthermore, the viscoelastic properties of the binders were evaluated over time by analyzing master curves for the relaxation modulus and creep compliance. These curves were generated by analyzing relaxation time spectra using the formula derived from the TTSP and GM models with the collected data.

From the laboratory experiments and subsequent analysis, the following specific conclusions can be drawn:

(1) For all binders, the dynamic modulus decreases as the temperature increases, while the phase angle increases. This suggests that the resistance to deformation of the binders decreases as the temperature increases, and the viscous properties become more prominent.
(2) Incorporating fibers into an asphalt binder can enhance its deformation resistance, as indicated by the data for dynamic modulus and phase angles. PPFs are the most effective additive for improving high-temperature deformation resistance, while LFs are superior for low-temperature crack resistance in asphalt binders. Additionally, incorporating fibers can decrease the material’s susceptibility to temperature and a more significant elastic characterization, particularly for LFs.
(3) Based on the tested and calculated data, it is clear that the GM model with 13 units serves as an efficient representation of the viscoelastic characteristics of the binders under consideration. This is attributed to the model’s exceptional precision and effectiveness during calculations.
(4) Incorporating fibers improves the long-term deformation resistance of asphalt binders, as demonstrated by data from the relaxation modulus and creep compliance master curves. Incorporation of lignin fibers is the most efficient approach to improve long-term properties, followed by polyester fibers and then polypropylene fibers.

In summary, the addition of fibers resulted in a significant improvement in deformation resistance at high temperatures and long-term deformation. Additionally, further investigation could provide new insights into the topic by exploring the optimal form and content, as well as the rheological properties of fiber-reinforced asphalt mixtures. 

## Figures and Tables

**Table 1:** Properties of the modified asphalt binder.

Parameter	Test Result	Requirement
Penetration at 25 °C/0.1 mm	58.6	50–60
Ductility at 5 °C/cm	23.8	≥20
Softening point/°C	79.1	≥60
Flash point (COC)/°C	365	≥230
Kinematic viscosity at 135 °C/m^2^/s	2.2	≤3
Solubility (trichloroethylene)/%	99.6	≥99

**Table 2:** Technical parameters of various fibers.

Fibers	Length (mm)	Diameter (μm)	Density (g/cm^3^)	Tensile Strength (MPa)	Melting Point (°C)	Moisture Content (%)	Oil Absorption (Times)
Lignin	5	-	1.10	-	-	-	6.2
Polypropylene	6	35.0	0.91	450	220	0.8	-
Polyester	8	24.0	1.38	508	260	1.0	-
Standard	6–12 (LFs < 6)	15–35	-	>270	>220	≤1.0	5–9

**Table 3:** Technical indicators of mineral powder.

Projects	Unit	Measurement Value	Specification Requirements
Apparent density	t/m^3^	2.694	1
Hydrophilic coefficient	—	0.76	<1
Size range < 0.6 mm	%	100	100
<0.15 mm	%	95.8	90~100
<0.075 mm	%	85.7	75~100

## Data Availability

No new data were created or analyzed in this study. Data sharing is not applicable to this article.
